# Wearable Sensors and the Evaluation of Physiological Performance in Elite Field Hockey Players

**DOI:** 10.3390/sports12050124

**Published:** 2024-04-29

**Authors:** Francesca Latino, Francesco Tafuri

**Affiliations:** 1 Department of Human Science, Educational and Sport, Pegaso University, 80100 Naples, Italy; 2 Heracle Lab Research in Educational Neuroscience, Niccolò Cusano University, 00100 Rome, Italy; francesco.tafuri@unicusano.it

**Keywords:** sensors, physical exercise, physiology

## Abstract

Sports performance tracking has gained a lot of interest and widespread use in recent years, especially in elite and sub-elite sports. This makes it possible to improve the effectiveness of training, to calibrate and balance workloads according to real energy expenditure, and to reduce the likelihood of injuries due to excessive physical stress. In this context, the aim of this review was to map the scientific literature on wearable devices used in field hockey, evaluating their characteristics and the available evidence on their validity in measuring physiological and movement parameters. A systematic investigation was carried out by employing five electronic databases and search terms that incorporated field hockey, wearables, and performance analysis. Two independent reviewers conducted assessments of the 3401 titles and abstracts for inclusion, and at the end of the screening process, 102 full texts were analyzed. Lastly, a total of 23 research articles that specifically concentrated on field hockey were incorporated. The selected papers dealt with performance monitoring (6 papers), technical analysis and strategy game (6), injury prevention (1), and physiological measurements (10). To appraise the quality of the evaluations, the Oxford quality scoring system scale was employed. The extraction of information was carried out through the utilization of the participants, intervention, comparison, and outcomes (PICOS) format. The analysis encompassed research studies that implemented wearable devices during training and competitive events. Among elite field hockey competitions, GPS units were identified as the predominant wearable, followed by heart rate monitors. The intraclass correlation coefficient (ICC) related to wearable devices showed reasonably high between-trial ICCs ranging from 0.77 to 0.99. The utilization of wearable devices in field hockey primarily centers around the measurement of player activity profiles and physiological demands. The presence of discrepancies in sampling rates and performance bands makes it arduous to draw comparisons between studies. Nevertheless, this analysis attested to the fact that wearable devices are being employed for diverse applications in the realm of field hockey.

## 1. Introduction

Over the past few decades, technologies have played a pivotal role in the transformation of sport, leading to a series of revolutions that have changed the way athletes compete and spectators experience sports [1]. One of the most influential innovations in sports is data analysis through wearable sensors. Wearable technology mainly refers to smart electronic devices that can be applied to clothing or directly to the body. They are tools capable of connecting and communicating with other devices, transmitting, monitoring and detecting information and data of all kinds starting from the vital signs of our body [2]. Furthermore, wearable sport devices offer several important features that facilitate data collection outside the laboratory and in more ecological environments. They play a crucial role in bringing laboratory-grade performance monitoring and analysis capabilities to the field, empowering athletes and coaches to optimize performance, prevent injuries, and achieve their goals in real-world, ecological environments [3]. In fact, wearable devices enable athletes to monitor their performance in real-world conditions, which often differ significantly from controlled laboratory settings. These real-world data offer a more accurate representation of an athlete’s abilities and allow for more effective training and performance optimization. By collecting data directly from outdoor activities, wearable devices provide contextual insights that are not possible to replicate in a laboratory setting [4]. For example, GPS watches can track elevation changes, heart rate variations, and pacing strategies during a trail run, offering valuable insights into how athletes perform in specific environmental conditions. In addition, natural environments present various challenges such as terrain changes, weather fluctuations, and altitude variations [5,6]. Wearable devices designed for outdoor use are built to withstand these challenges, providing reliable data even in harsh conditions. This adaptability ensures that athletes can rely on their devices regardless of the environment they are training or competing in. Therefore, the ability of wearable sport devices to capture data in ecological environments is essential for providing athletes with accurate, context-specific insights into their performance, enhancing safety and optimizing training strategies for outdoor activities [7].

It is for these reasons that sports performance tracking has gained a lot of interest and widespread use in recent years, especially in elite and sub-elite sports [8]. The goal of wearable devices is to quantitatively evaluate the athlete’s performance during training or matches and to choose and optimize the training strategy. This makes it possible to improve the effectiveness of training, to calibrate and balance workloads according to real energy expenditure, and to reduce the likelihood of injuries due to excessive physical stress [9].

Today, high-level professional clubs across different sports routinely employ performance measurement technologies through wearable sensors, capturing parameters such as the position, speed, distance, accelerations, and change in direction of each athlete. From this data, other relevant information can be calculated retrospectively (i.e., after the training session), such as metabolic load, speed/acceleration thresholds, change in direction, sprints, total distance, etc. Advanced devices, designed specifically to optimize performance, provide detailed biometric and analytical information to take athletic performance to the next level [10]. Wearable devices have thus established themselves as indispensable tools for equipping competitive athletes [11]. Their ability to capture accurate biometric data and quantify training performance helps athletes train in a smarter way [10]. Wearables allow for the following:

i. In-depth knowledge of physiology: advanced sensors monitor key parameters such as heart rate variability (HRV) and oxygen saturation (SpO_2_) to optimize training response [12];
ii. Fatigue and recovery analysis: analyses assess training readiness and adjust training load in order to balance fitness and prevent injury [13];
iii. Training and technique optimization: detailed statistics, such as running dynamics, impact frequency, jump height, etc., provide athletes with feedback to improve their form [14];
iv. Comprehensive quantitative analysis: hundreds of aggregated data points by week, month, and year allow for fine-tuning and customization that would be unthinkable without wearables [15].

Increasingly, these sensor sets incorporate sports performance meters, the variety of which has grown over time [16,17]. Among the most widespread technologies are the following:

i. Motion trackers or motion capture used to study the kinematics of movement;
ii. Slow-motion cameras used for the analysis and improvement of athletic gestures;
iii. Cameras for thermographic analysis, i.e., the real-time measurement of body temperature, in order to prevent possible injuries and better understand phenomena such as vasoconstriction;
iv. Systems to analyze the reaction of the body under stress, which can be combined with electrocardiogram, pressure measurement, and spirometry analysis, to better understand the exchange of intrapulmonary gases;
v. Electromyographs, instruments that allow athletes to record and analyze the electrical signal generated by muscles during their contraction;
vi. Metabolimeters, for the evaluation of athletes in types of movement such as running, rowing, and pedaling, both on competition equipment and in training.

In recent years, the interest in the use of wearable devices capable of quantitatively measuring the athletic performance carried out appears to be growing considerably in field hockey [18]. The physical condition of field hockey players is significant to both their personal performance and the collective effectiveness of the team, because of the fast- and intermittently paced nature of the sport. Given the diverse applications of wearable devices, it would be intriguing to acquire a comprehensive understanding of their utilization in the context of field hockey. Field hockey exhibits dissimilarity compared to other sports due to its allowance of unrestricted substitutions among players, giving rise to a unique set of physical requirements specific to the sport and distinct from those encountered in other athletic endeavors [19,20].

The ability to obtain and analyze a good quantity of physiological information has changed the way of understanding the training of both the team and the individual. Athletes, thanks to sensors, provide the coach with data that allow for the monitoring of performance, health, fatigue, and stress. This is very important information that helps coaches make certain choices regarding training and competition [21].

Therefore, the aim of this review was to map the scientific literature on wearable devices used in field hockey, evaluating their characteristics and the available evidence on their validity in measuring physiological and movement parameters.

### 1.1. Wearable Categories

In the past years, a lot of experience has been acquired with wearable-based performance measurement instruments, especially video analysis and GPS [22]. These technologies have proven to be a simple and effective way to capture and measure relevant player data. In recent years, however, the availability of miniaturized and precise inertial sensors (especially accelerometers and gyroscopes) has greatly expanded the possibility of monitoring sports performance, making alternative and even-more-advanced approaches possible. The following sections will explain the difference between the three technologies, highlighting the possibilities and limitations, as well as the pros and cons of each approach, through an in-depth review of the most significant research in the literature [23].

#### 1.1.1. Video Analysis

Sports video analysis involves the utilization of video footage to meticulously examine and assess the performance of athletes. It can be most effectively comprehended as the comprehensive process of dissecting an individual’s performance in a particular sport, typically on video [24]. This potent technology empowers coaches and players with informative insights, enabling them to make precise adjustments to their technique to attain peak performance, thereby proving to be an invaluable educational resource. By utilizing specialized software and data tracking tools, it is now feasible to isolate and scrutinize crucial elements of performance with remarkable agility and precision. In fact, the use of wearables can greatly enhance video analysis by providing additional data points and context to the analysis process and serving as an educational resource.

There are various forms of sports performance analysis in which wearables can be used in conjunction with video analysis. These encompass the following:

− Biometric data collection: wearables like fitness trackers or smartwatches can collect biometric data such as heart rate, blood pressure, and body temperature. These data can be synchronized with video footage to analyze an individual’s physiological responses during certain activities or events captured on video [25];
− Motion tracking: wearable motion sensors, such as accelerometers and gyroscopes, can track movement and orientation in real-time. When synchronized with video footage, these data can provide precise information about body movements, posture, and gestures, enabling detailed analysis of athletic performance, physical therapy exercises, or ergonomic evaluations [25];
− Point-of-view cameras: wearable cameras, such as body-worn action cameras or smart glasses, capture video from the wearer’s perspective. These devices can provide unique insights into first-person experiences, allowing coaches to understand the wearer’s viewpoint and reactions in various situations, such as in training and competitions [26];
− Contextual information: wearables with biometric sensors and machine learning algorithms can analyze athletes’ behavior patterns over time. By correlating these data with video recordings, coaches can gain insights into athletes’ habits, preferences, and performance trends, facilitating personalized coaching [26];
− Real-time feedback: wearables with built-in feedback mechanisms, such as haptic alerts or audio cues, can provide real-time guidance or notifications based on video analysis results. For instance, in rehabilitation scenarios, wearable devices can alert athletes when they deviate from prescribed movements, when captured on video, helping them correct their form and prevent injuries [26];

Training: wearables can enhance training programs by combining video analysis with interactive learning experiences. For example, athletes can wear smart simulation devices that record their actions during simulated procedures, while coaches analyze the video footage to provide personalized feedback and guidance [27].

Therefore, integrating wearables with video analysis expands the scope and depth of insights that can be gleaned from visual data, enabling applications across various domains, including sports performance analysis and the prevention of injuries.

#### 1.1.2. Global Positioning Systems (GPS)

The GPS officially known as NAVSTAR GPS (NAVigation Satellite Time and Ranging Global Positioning System) is under the ownership of the United States government. As of now, this system comprises a total of 24 operational satellites. GPS operates under any weather conditions, on a global scale, around the clock and does not entail any charges for membership or installation. The United States Department of Defense (USDOD) originally launched satellites into space with the intention of serving military objectives [28]. However, during the 1980s, these satellites were rendered accessible for civilian applications. GPS satellites orbit the Earth in a regular manner, completing one revolution in approximately 12 h. These satellites emit a distinct signal that contains specific orbital elements. This signal enables GPS devices to decipher the information and determine the exact location of the satellite in space. GPS receivers utilize these data and employ the trilateration technique to compute the precise geographical coordinates of a user’s position on the Earth’s surface [29]. The GPS receiver gauges the distance to each satellite by assessing the time required to receive a transmitted signal. By conducting numerous measurements, it can ascertain its own position and present it on a screen to quantify, for instance, the trajectory of a run [30].

Today, GPS is built into virtually everything, in smartwatches, smartphones, and satellite communicators, and can be found installed in cars, boats, and more. To compute the two-dimensional location, encompassing latitude and longitude, and monitor the motion, it is imperative to connect a Global Positioning System (GPS) receiver to a minimum of three satellites. By having four or more satellites within sight, the receiver can ascertain the three-dimensional position, encompassing latitude, longitude, and altitude. In general, a GPS receiver can identify a minimum of eight celestial bodies simultaneously, with the precise number contingent on the specific temporal conditions and one’s geographical coordinates on the terrestrial plane [31].

GPS technology can offer valuable insights in field hockey, enhancing performance analysis, training, and strategic decision making. An alternative possibility for wearable technology is the adoption of the Local Positioning System (LPS) instead of the conventional GPS [32], which enables the quantification of activity profiles. LPS operates in a comparable manner to GPS, with the exception that base stations, rather than satellites, are deployed throughout the field hockey field. Athletes are subsequently mandated to employ active transponders affixed to chest straps, enabling the transmission of signals to the aforementioned base stations. Since there are numerous activities that the player performs in a given technical–tactical exercise, as evidenced by the performance model provided by the video analysis, the GPS provides a series of parameters that can be grouped into the following: position on the pitch; velocity; acceleration; distance; and metabolic power. The main objectives for which it is useful to use GPS in field hockey are the following:

1. Study the performance model. Through race tracking, GPS offers mean reference values, that is, all the actions performed by the player throughout the race and in their designated position [33];
2. Load Management. GPS data provide insights into players’ physiological loads during training sessions and matches. Coaches can monitor metrics like distance traveled, high-speed running, and sprint efforts to ensure players are not overexerted and to tailor training programs for optimal physical conditioning and recovery. GPS technology enables individuals to govern the intensity of their training session, microcycle, and mesocycle. This is particularly valuable for activities involving a ball, as it offers insights into external exertion. It grants the opportunity to assess whether the predetermined objectives have been accomplished, as well as if specific exercises impose an adequate physical strain and what their ultimate purpose is. Moreover, it facilitates the monitoring of players both over an extended period and across different dimensions. It also evaluates the players’ movements [34];
3. Evaluate the technical–tactical exercises. The exercises are examined by means of calculating the averages and are arranged into categories based on their respective types. Consequently, it becomes feasible to obtain an overview of the workload of the exercises, considering both the type of exercise and the governing principles that dictate and regulate the workload. Furthermore, it becomes feasible to assess whether a suggested exercise primarily elicits a metabolic or neuromuscular response [35];
4. Evaluate small-sided games. In addition to the technical–tactical components, small-sided games stimulate, above all, the specific physical ones. GPS provides the loads and objectives of such exercises. Technical–tactical circuits with physical targets are also evaluated based on their modulation. Although GPS technology is not as precise as video analysis, it is still regarded as the most advantageous method for monitoring sports performance. [36].

#### 1.1.3. Inertial Sensors

The most advanced athlete monitoring technologies make it possible to integrate the use of GPS with motion sensors. A motion sensor returns the metrics collected by an inertial sensor, a device capable of measuring variables of the movement of a body to which it is physically attached, such as accelerations, decelerations, displacements, jerks, speeds, shocks absorbed, and falls. There are three types of inertial sensors, accelerometers, gyroscopes, and magnetometers. Accelerometers provide a measure of the linear acceleration to which the body is subjected. Gyroscopes measure angular velocity. Magnetometers are advanced compasses capable of indicating the direction of Earth’s magnetic north [37]. The extreme degree of miniaturization achieved with MEMS (Micro Electro–Mechanical Systems) technology, used to build the sensors, has made it possible to steadily reduce costs, greatly favoring their diffusion. A primary differentiation among inertial sensors lies in the quantity of susceptible axes, specifically the axes upon which the measurement is conducted with variables ranging from 1 to 3. To illustrate, in order to quantify a swimmer’s acceleration, a single-axis accelerometer, positioned along the direction of progression, is required. In the case of three-dimensional movements such as those made by a footballer moving in different directions, a triaxial sensor will be required. The first prototypes of sensors used in sports date back to the 2000s [38]. Today, there are sensors on the market that integrate accelerometers, magnetometers, and gyroscopes. Accelerometers are also available that integrate gyroscopes with pressure sensors (altimeters), acoustic sensors (microphones), and direction sensors (magnetometers). It is a “superset” of instruments capable of returning complex measurements thanks to the integration in modules (System-in-Package) of inertial sensors, with up to nine axes. They house the microelectronics necessary to support data processing, data fusion algorithms based on Kalman filtering, and machine learning [39].

## 2. Materials and Methods

### 2.1. Search Strategy

This systematic review has been compiled adhering to the guidelines set forth by the Preferred Reporting Items for Systematic Reviews and Meta-Analyses (PRISMA) statement. [40]. Five academic databases, PubMed, Google Scholar, Cochrane Library, Embase, and Web of Science, were systematically searched to identify original research studies. They had to be in the English language and to have undergone peer review in order to explore the validity and/or reliability of microtechnology embedded in wearable devices. These studies aim to map the scientific literature on wearable devices used in field hockey, evaluating their characteristics and the available evidence on their validity in measuring physiological and movement parameters. Studies were identified through the utilization of the subsequent Boolean search syntax: “((wearable sensors or “wearable devices”) and (“field hockey” or “hockey”)”/“(“intermittent team sport”) and (performance analysis))”. After completing the initial steps, the subsequent filters were put into effect, including the availability of complete text, restriction to human research, requirement for the language to be English, and a limitation to articles published within the last fifteen years. The methodology utilized for searching the PubMed database involved a combination of the MeSH database and Boolean search syntax. Once a set of potential articles was gathered, the next stage involved further refinement based on predetermined criteria for inclusion and exclusion. The software tool Zotero 5.0.85 was used to scrutinize the records for any occurrences of replication. All potential variations in spelling, encompassing the truncated search term implementing wildcard symbols, together with the application of the “related articles” functionality, were employed in combination with the Boolean operators AND and OR. Two independent reviewers conducted assessments of the titles and abstracts of all studies identified through database exploration. The complete text of potentially relevant articles was evaluated to determine their eligibility. Only original research articles published in the English language were considered. In cases where there was a disagreement regarding the inclusion of a study, a third reviewer was consulted. Following the collection of candidate articles, additional screening was conducted based on the predetermined criteria for inclusion and exclusion. Estimating inter-rater reliability was performed using Cohen’s Kappa (k = 0.61).

### 2.2. Selection Criteria

Duplicate studies were eliminated, and the titles and abstracts of all the remaining investigations were selected based on relevance according to two scholars. Papers that were considered to be outside the purview of the analysis were excluded. The complete texts of the remaining investigations were subsequently evaluated for suitability. Eligibility criteria of the studies were based on the PICOS (Population, Intervention, Comparison, Outcomes, and Study design) framework [41]. Thus, the selection criteria and search strategy were designed to answer the above-mentioned research questions. If the studies provided relevant information concerning the PICOS system and met the predetermined requirements for inclusion, they were considered appropriate for incorporation. The criteria for inclusion were as follows (Table 1):

If it was determined that a study did not fulfill the criteria for inclusion, or lacked a focus on field hockey, the study was excluded. Moreover, review articles, meta-analyses, and unpublished studies were excluded from the analysis. Subsequently, a manual search was conducted on the reference list of all eligible studies to identify any studies that were not found during the initial search. If any such study was discovered, it underwent the same evaluation process as previously explained. This study did not consider review articles, meta-analyses, and unpublished studies. Nevertheless, these sources were consulted as a means of reference to determine the initial search and evaluate their suitability for incorporation.

## 3. Results

### 3.1. Identification of Studies

**Figure 1:**
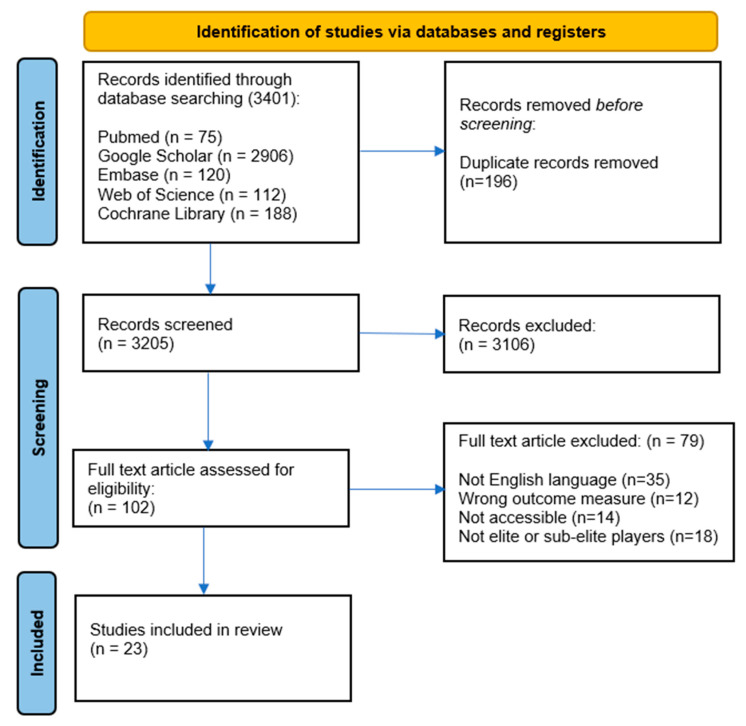
The study selection and eligibility screening flow according to PRISMA guidelines.

The systematic inquiry acquired a sum of 3401 manuscripts, of which 196 were eliminated as duplicates. Thus, titles and abstracts of the remaining 3205 papers were evaluated, and subsequently, 3106 were considered to be clearly beyond the scope of the analysis. In fact, they explored variables that were not of fundamental interest for this study. Consequently, they were eliminated, and the complete document of the remaining 102 investigations was evaluated. Subsequently, it was determined that 23 investigations met the inclusion criteria and were included in this analysis. The process of identification is delineated in Figure 1.

### 3.2. Quality Assessment

The evaluation of the standard of all the studies was carried out by utilizing two distinct assessment instruments: the Oxford quality scoring system scale and the Risk-of-Bias assessment graph. The Oxford quality scoring system scale was used to selectively determine the quality of the studies in a subjective manner (Table 2). This instrument was employed to assess the methodological excellence of the chosen studies that were included in this comprehensive review. This approach is considered acceptable in evaluating the methodological integrity of observational study designs and has been previously employed by systematic reviews in the field of sport science [42].

**Figure 2:**
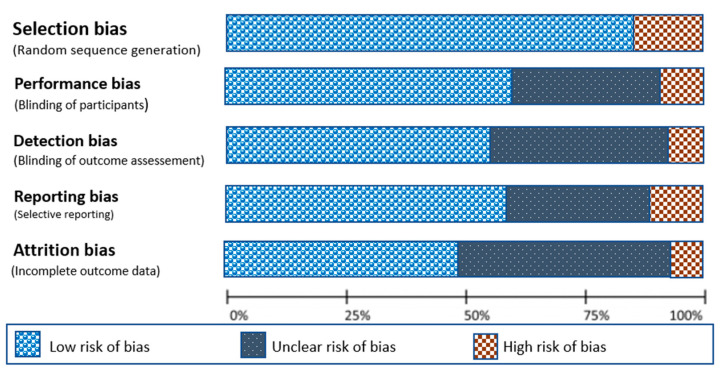
Risk-of-bias assessment.

As suggested by Cochrane [66] for the assessment of bias risk, an excel RoB2 graph was utilized (Figure 2). This tool enables an emphasis on transparency and methodo-logical rigor and was implemented for every study that was incorporated.

### 3.3. Study Characteristics

This review considered an analysis of 23 high-quality studies focused on the validity and reliability of wearable microtechnology. The summary characteristics of reviewed studies are listed in Table 3.

## 4. Discussion

The results of this systematic review revealed that sensor devices can play a significant role in field hockey by providing valuable data and insights to players, coaches, and sports scientists. They are frequently employed to assess player profile and physiological requirements.

All studies included in this review utilized valid and reliable wearable devices. In fact, the intraclass correlation coefficient (ICC) related to wearable devices showed reasonably high between-trial ICCs ranging from 0.77 to 0.99. The different ways through which sensor devices can enhance the effectiveness of field hockey include the following: performance monitoring (player tracking and team analysis), technical skills analysis (stick and ball movement, accuracy, and power), tactical insight (game strategy), injury prevention (biomechanical analysis), gaming optimization (workload management), and physiological measurements (heart rate and rating of perceived exertion).

### 4.1. Performance Monitoring

Regarding performance monitoring, sensors can be attached to players’ uniforms or equipment to monitor their movements during a game or practice. These data can include distance covered, speed, acceleration, and deceleration. This information can be crucial for assessing a player’s physical condition and performance on the field [67,68,69,70]. Coaches can use this information to analyze player performance and make data-driven decisions. Sensors can also track the positioning and movements of the entire team. These data help coaches identify patterns, assess team coordination, and develop strategic game plans. In studies examining the profiles of player activity, the parameter that was most frequently assessed was distance, as it was recorded by all studies utilizing the technology of GPS [71,72,73]. Subsequently, the analysis considered speed, which was further divided into two categories: the speed associated with locomotor activities (walking, running, and sprinting) and the speed associated with running intensity (low, moderate, and high levels). Despite the similarities in the variables that were measured, the different activities considered create challenges when attempting to compare different studies [49,51,55]. In 2015, Polglaze and colleagues [44] in their study utilized the MiniMax Catapul 10 Hz, a valid and reliable device (ICC > 0.90). The authors found a significant correlation between the distance covered and the performance level in the realm of elite men’s hockey. However, it should be noted that there is a degree of variability observed between competitive matches and training sessions, as well as among different player positions. This suggests that the accumulation of player load in the sport of hockey is predominantly attributed to running and other forms of locomotion. Consequently, player load is not efficacious in measuring other activities such as evasion and assuming a low stance, which significantly contribute to the physiological demands, especially during training. Equally interesting is the research by McGuiness et al. [62] in which they used Statsports—APEX Athletes, an excellent device that showed significant validity and reliability (ICC > 0.96). In their study, the researchers measured the length of time and the exact location of peak running capability in a group of 31 highly skilled female field hockey players during actual game situations. The authors demonstrated that the forwards exhibited the most superior running capabilities throughout all time intervals due to their strategic responsibilities within the outlet phases of play in dynamic leading running. Furthermore, the participation of forwards in counter-attacking play led to heightened running requirements in comparison to their counterparts. The study by Sunderland et al. [46] elucidated the activity profile of elite male field hockey players over the course of two seasons, thanks to the use of a highly reliable device, namely SpiElite—GPSports (ICC > 0.95). The study was groundbreaking as it was the first to document match-to-match variability and assess the impact of the implementation of the self-pass rule on the activity profile. The duration of play differed significantly depending on the position held, thereby influencing the activity profile. Fullbacks exhibited a higher total distance covered, whereas forwards surpassed all other positions in terms of mean speed, percentage of time spent engaging in high-speed running, and sprinting. The study by Sunderland and colleagues illustrated that the profile of activity, and by extension, the physical requirements imposed on athletes, exhibit significant variations according to their respective playing positions. The average velocity exhibits an upward trend as one moves from fullbacks and halfbacks to midfield, and further increases for forwards. This observation aligns with the findings of other contemporary studies investigating the physical demands associated with various positions in professional men’s field hockey.

### 4.2. Analysis of the Technical Skills and Game Strategy

Another factor commonly evaluated within the included studies concerns the analysis of the technical skills of field hockey players. The progression of technology and the perpetual requirement for augmenting performance in athletic activities [74] have expedited the application of wearable sensors in the domain of sports data analysis [75]. These devices have also been utilized in the context of field hockey, such as in the identification of players’ actions and the enhancement of their skills [48]. In fact, sensors can be attached to hockey sticks and balls to capture data on stick handling, passing, and shooting techniques. This information can be used to analyze the technical skills of individual players and the team as a whole. In addition, sensors can measure the accuracy and power of shots, allowing players to refine their skills and allowing coaches to tailor training programs to address specific weaknesses. Morencos and colleagues [57] conducted a study that investigated a sensor-based methodology to enhance the fundamentals of play. Thanks to the use of Spi Elite—GPSports (ICC > 0.95), several sensors were utilized at various points of contact on the stick and were linked to a range of auditory feedback cues. This allowed players to instantaneously receive feedback on the efficacy of their technical skills. Moreover, additional research has concentrated on the examination of technical proficiencies. Furthermore, body sensors were employed to aid novice athletes in accomplishing a triumphant drag-flick. The study’s participants have conveyed enhancements in drag-flick methodology, a reduction in bodily strain while executing the shot, and have praised the heightened level of engagement in the learning process.

This revision also makes it possible to include wearable sensors as indispensable tools for the analysis of game strategy. Sensors, in fact, can contribute to the analysis of game strategies and tactics, providing valuable data that can be used to enhance player performance, optimize team strategies, and improve overall gameplay [59]. Coaches can use data on player positioning, ball movement, and other relevant factors to adjust their game plans and exploit opponents’ weaknesses. For instance, geographical positioning system (GPS) data have been employed to establish correlations among various playing positions, game halves and quarters, age cohorts, levels of competition, and even among elite field hockey matches.

### 4.3. Injury Prevention

With regard to injury prevention, wearable sensors can provide insights into players’ biomechanics, helping identify any irregularities in movement patterns that may lead to injuries. Coaches and medical staff can use this information to design injury prevention programs and modify training regimens [76]. In fact, in the studies examined, GPS technology has also been employed to evaluate the susceptibility of players to sustain injuries. The research conducted by Kim et al. [50] explored the correlation between activities undertaken by players during competitions and the occurrence of non-contact ankle and knee injuries (Spi Elite—GPSports; ICC > 95). Furthermore, in their study, Warman et al. [56] utilized MiniMaxX—Catapult (ICC > 90) devices to examine postural data, specifically focusing on the angles of torso flexion and extension in elite male field hockey players during competitive matches. These instances serve to demonstrate the wide array of applications in which GPS technology has been utilized to observe the postural requirements and injury-related variables of field hockey players.

### 4.4. Physiological Measurements and Workload Management

When it comes to analyzing physiological measurements and workload management, sensors can help monitor players’ workloads during training sessions to prevent overtraining and reduce the risk of injuries. This is particularly important in a sport like field hockey, where high-intensity sprints and directional changes are common [77]. Some sensor devices provide real-time data, allowing players and coaches to receive immediate feedback during training sessions or games. This can be valuable for making quick adjustments and improvements [45,52]. Heart rate data were employed in various inquiries, either for the purpose of conducting comparisons amidst different gender and age groups, or for the purpose of computing training loads to establish correlations between heart rate and other factors, such as the rate at which exertion is perceived. Monitoring players’ heart rates during a game can provide insights into their cardiovascular fitness and help in managing fatigue [54]. These data can be used to optimize player substitution and rest periods. The application of GPS and heart rate monitors provided additional examinations concerning the physiological requirements and burdens experienced by the athletes throughout their matches [60,62]. The adoption of GPS and heart rate monitors revealed that warm-up exercises significantly contribute to the overall physical and physiological demands during the games [54,58,59].

Surprisingly, the study that stands out the most among the others is the one conducted by Malan et al. [43], in which, through the use of a valid and reliable devise (ICC > 96), such as Polar, the authors investigated the thermoregulation responses of elite field hockey goalkeepers. These goalkeepers were instructed to wear a sensor device positioned at the posterior of their pelvic guards following the ingestion of a radio-telemetry pill. Remarkably, this study is the sole one that focuses on the examination of goalkeepers rather than outfield players, thereby highlighting the scarcity of research on goalkeeper performances during competitive events. Goalkeepers are often omitted from activity profile analyses due to the belief that their running distances are insufficient for performance evaluation. Nevertheless, given their low probability of being substituted during a game, it is crucial to monitor their performances during competitions to mitigate the potential for injury and to facilitate the development of suitable training plans. In the study conducted by Malan et al. [61], it was observed that the core temperatures of the goalkeepers experienced an increase both prior to and following games under mild environmental circumstances. Considering the necessity for goalkeepers to wear protective goalkeeping equipment, knowledge regarding their core temperatures can prove valuable to coaches, allowing them to ensure that goalkeepers maintain proper hydration levels, particularly in environments characterized by high heat and humidity.

In recent years, Perrotta and his colleagues [47,53] undertook an examination to determine the extent of correlation between a universal indicator of exercise stress, which was obtained through ratings of perceived exertion (RPE), and a training load derived from heart rate (HR) during distinct stages of a competitive mesocycle in elite field hockey athletes (HRMonitor—Polar; ICC > 0.99). Their research brings forth a fresh perspective on the different levels of correlation between the subjective RPE and the training load derived from HR during specific stages of a mesocycle in field hockey players of elite status.

Beyond GPS and heart rate monitoring, which constitute the two most frequently employed apparatuses for evaluating the performance of players and teams during field hockey matches, additional metrics including core temperature, hydration status, skin temperature, hydration levels, and muscle fatigue are also subjects of considerable interest. These data can be used to prevent injuries and optimize training regimens. Therefore, it is imperative for engineers and researchers to persist in their efforts to generate groundbreaking wearable technologies with the aim of furnishing comprehensive insights into the overall performance, welfare, and safety of players.

## 5. Conclusions

Research on wearable devices in field hockey is steadily growing, aiming to enhance performance analysis, injury prevention, and player monitoring in field hockey. While more research is needed to validate their effectiveness fully, current evidence suggests that these devices provide valuable insights into players’ physiological and movement parameters, contributing to optimized training programs and strategies.

Overall, wearable sensors offer practical implications that extend beyond traditional coaching methods in field hockey. By harnessing the power of data-driven insights, coaches can make informed decisions, maximize player potential, and elevate team performance to new heights. The findings of this review demonstrated that wearables are widely utilized in field hockey to assess player profile activities (via GPS) and physiological requirements (via heart rate monitors). It is noteworthy to highlight the myriad of potential applications that can stem from data derived from these wearable devices. Specifically, GPS data can be leveraged to draw comparisons among various factors such as playing positions, match halves and quarters, age categories, and competition levels. Likewise, heart rate data find their use either for comparative purposes across different age groups or for computing training burdens to establish correlations between heart rate and other parameters like rate of perceived exertion. The combination of GPS and heart rate monitors facilitates supplementary analyses on the physiological demands and burdens encountered by athletes during their matches. Although GPS and heart rate monitors are the predominant devices in evaluating player and team performance in field hockey competitions, there is also a keen interest in exploring additional metrics such as core temperature, hydration levels, and susceptibility to injuries. It is imperative for engineers and researchers to persist in developing cutting-edge wearables to furnish comprehensive insights into players’ overall performance, welfare, and safety.

## Figures and Tables

**Table 1:** PICOS eligibility criteria.

POPULATION	− Elite and sub-elite male and female field hockey.
INTERVENTION	− Intervention takes place in the context of field hockey; − Aim of the intervention is to improve any mental health or − cognitive outcome of students.
CONTROL	− Presence of a parallel control group.
OUTCOME	− Performance monitoring; − Analysis of the technical skills and game strategy; − Injury prevention (biomechanical analysis); − Physiological measurements and workload management; − Assessed the validity and/or reliability of wearable micro − technology to quantify movement or specific actions.
STUDY DESIGN	− Study design must be RCT or crossover RCT with a parallel − control group; − Study design must include true randomization; − Published in a peer-reviewed academic journal; − English-language publications; − Time interval of studies between 2009 and 2024.

**Table 2:** The Oxford quality scoring system scale.

Authors	Was the Treatment Randomly Allocated?	Was the Randomization Procedure Described and Was It Appropriate?	Was There a Description of Withdrawals and Dropout?	Was There a Clear Description of the Inclusion/Exclusion Criteria?	Were the Methods of Statistical Analysis Described?	Jadad Score (0–5)
Malan et al., (2010) [43]	No	No	Yes	Yes	Yes	3
Polglaze et al., (2015) [44]	Yes	No	Yes	Yes	Yes	4
Vescovi et al., (2016) [45]	No	No	Yes	Yes	Yes	3
Sunderland et al., (2017) [46]	Yes	No	Yes	Yes	Yes	4
Perrotta et al., (2017) [47]	Yes	Yes	Yes	Yes	Yes	5
Morencos et al., (2018) [48]	Yes	Yes	Yes	Yes	Yes	5
Casamichana et al., (2018) [49]	No	No	Yes	Yes	Yes	3
Kim et al.(2018) [50]	Yes	Yes	Yes	Yes	Yes	5
Vinson et al.(2018) [51]	No	No	Yes	Yes	Yes	3
Vescovi et al. (2018) [52]	Yes	Yes	Yes	Yes	Yes	5
Perrotta et al., (2018) [53]	Yes	Yes	Yes	Yes	Yes	5
Polglaze et al., (2018) [54]	Yes	Yes	Yes	Yes	Yes	5
Chesher et al., (2019) [55]	Yes	No	Yes	Yes	Yes	4
Warman et al., (2019) [56]	Yes	No	Yes	Yes	Yes	4
Morencos et al., (2019) [57]	Yes	No	Yes	Yes	Yes	4
McGuinness et al., (2019) [58]	Yes	No	Yes	Yes	Yes	4
Harry et al., (2020) [59]	No	No	Yes	Yes	Yes	3
Romero-Moraleda et al., (2020) [60]	No	No	Yes	Yes	Yes	3
McGuinness et al., (2020) [61]	Yes	No	Yes	Yes	Yes	4
McGuinness et al., (2021) [62]	Yes	Yes	Yes	Yes	Yes	5
Ihsan et al., (2021) [63]	Yes	Yes	Yes	Yes	Yes	5
McGuinness et al., (2022) [64]	Yes	Yes	Yes	Yes	Yes	5
Lin et al., (2023) [65]	Yes	No	Yes	Yes	Yes	4

**Table 3:** Summary characteristics of reviewed studies.

Category of Studies	Authors	Validity/Reliability	Sample	Device	Measures
GPS	Polglaze et al., (2015) [44]	ICC > 0.90	24 elite male field hockey players	MiniMaxX—Catapult	Relationships between distance and player load
	Vescovi et al., (2016) [45]	ICC > 0.95	68 elite male field hockey players	Spi Elite—GPSports	Monitoring locomotor demands and metabolic-power characteristics
	Sunderland et al., (2017) [46]	ICC > 0.95	28 elite male field hockey players	Spi Elite—GPSports	Position-specific activity profile
	Morencos et al., (2018) [48]	ICC > 0.95	16 elite male field hockey players	Spi Elite—GPSports	Influence of the match period on the movement patterns
	Casamichana et al., (2018) [49]	ICC > 0.95	16 elite male field hockey players	Spi Elite—GPSports	Monitoring running demands of professional field hockey players
	Kim et al.(2018) [50]	ICC > 0.95	32 elite female field hockey players	Spi Elite—GPSports	Assessing injuries to the lower extremities
	Vinson et al.(2018) [51]	ICC > 0.95	6 women’s field hockey teams	Spi Elite—GPSports	Assessing relative distance covered by the various playing positions
	Chesher et al., (2019) [55]	ICC > 0.90	15 elite male field hockey players	MiniMaxX—Catapult	Monitoring deceleration efforts
	Warman et al., (2019) [56]	ICC > 0.90	16 elite male field hockey players	MiniMaxX—Catapult	Understanding the nature of a player’s torso postures in field hockey match-play, and its relationship with the perceptuomotor demands of the sport
	Morencos et al., (2019) [57]	ICC > 0.95	16 elite female field hockey players	Spi Elite—GPSports	Monitoring kinematic demands and technical skills
	McGuinness et al., (2019) [58]	ICC 0.98	27 elite female field hockey players	Vxsports—GPSports	Monitoring physical and physiological demands
	Romero-Moraleda et al. (2020) [60]	ICC > 0.95	10 elite male field hockey players	Spi Elite—GPSports	Monitoring physical demands profile
	McGuinness et al. (2020) [61]	ICC > 0.77	16 elite female field hockey players	OptimEye S5—Catapult	Monitoring wellness, training load, and running performance
	McGuinness et al., (2021) [62]	ICC > 0.96	28 elite male field hockey players	Statsports—APEX Athletes	Quantifying the rotational demands with respect to position
	Ihsan et al. (2021) [63]	ICC > 0.90	28 elite male field hockey players	MiniMax Team 2.5—Catapult	Monitoring running demands and activity profile
HR Monitor	Perrotta et al., (2017) [47]	ICC > 0.99	37 elite male field hockey players	HR Monitor—Polar	Relationship and validity between a vagal-related HRV index and rMSSD
	Vescovi et al., (2018) [52]	ICC > 0.99	1 women’s field hockey team	HR Monitor—Polar	Exploring differences between matches for warm-up and total session demands
	Perrotta et al., (2018) [53]	ICC > 0.99	17 elite male field hockey players	HR Monitor—Polar	Monitoring magnitude of correlation between ratings of perceived exertion and time spent above threshold and two HR-derived training loads
	Polglaze et al., (2018) [54]	ICC > 0.99	16 elite male field hockey players	HR Monitor—Polar	Monitoring metabolic power characteristics
	Harry et al. (2020) [59]	ICC > 0.85	32 elite female field hockey players	Zephyr—HR Monitor	Monitoring physical match demands
Inertial Sensors	Malan et al. (2010) [43]	ICC > 0.99	6 elite male and female field hockey players	Polar	Examining the thermoregulatory responses in field hockey goalkeepers
	Lin et al., (2023) [65]	ICC > 0.84	18 male field hockey players	Vector S7—Catapult Sports	Investigating the peak running, mechanical, and physiological demands
	McGuinness et al., (2022) [64]	ICC > 0.83	23 female field hockey players	Johan Sports	Monitoring running performance

## References

[1] Livingston L.A., Cunningham I., Forbes S.L. (2023). Using technological innovation to manage and develop sport officials. Manag. Sport Leis..

[2] Seçkin A., Ateş B., Seçkin M. (2023). Review on Wearable Technology in Sports: Concepts, Challenges and Opportunities. Appl. Sci..

[3] Aidar F.J., Cataldi S., Badicu G., Silva A.F., Clemente F.M., Latino F., Greco G., Fischetti F. (2022). Paralympic Powerlifting as a Sustainable Way to Improve Strength in Athletes with Spinal Cord Injury and Other Disabilities. Sustainability.

[4] Latino F., Saraiello E., Tafuri F. (2023). Outdoor Physical Activity: A Training Method for Learning in an Experiential and Innovative Way. J. Phys. Educ. Sport.

[5] Morsanuto S., Cassese F.P., Tafuri F., Tafuri D. (2023). Outdoor Education, Integrated Soccer Activities, and Learning in Children with Autism Spectrum Disorder: A Project Aimed at Achieving the Sustainable Development Goals of the 2030 Agenda. Sustainability.

[6] Tafuri F., Latino F. (2024). School Medical Service: Strategies to Promote Psycho-Physiological Well-Being. Pediatr. Rep..

[7] Latino F., Tafuri F. (2024). Physical Activity and Cognitive Functioning. Medicina.

[8] Miah A. Rethinking enhancement in sport. In The Ethics of Sports Technologies and Human Enhancement; Routledge, London, UK, 2020; pp. 233–252.

[9] Luczak T., Burch R., Lewis E., Chander H., Ball J. (2019). State-of-the-art review of athletic wearable technology: What 113 strength and conditioning coaches and athletic trainers from the USA said about technology in sports. Int. J. Sports Sci. Coach..

[10] Liebermann D.G., Katz L., Hughes M.D., Bartlett R.M., McClements J., Franks I.M. (2002). Advances in the application of information technology to sport performance. J. Sports Sci..

[11] Omoregie P.O. The impact of technology on sport performance. Proceedings of the INCEDI 2016 Conference.

[12] Magdalinski T. (2019). Sport, Technology and the Body: The Nature of Performance.

[13] Benson L.C., Räisänen A.M., Volkova V.G., Pasanen K., Emery C.A. (2020). Workload a-WEAR-ness: Monitoring Workload in Team Sports with Wearable Technology. A Scoping Review. J. Orthop. Sports Phys. Ther..

[14] Carling C., Reilly T., Williams A.M. (2008). Performance Assessment for Field Sports.

[15] Gulhane T. (2014). Various Types of Advanced Technologies in Sports. IOSR J. Sports Phys. Educ..

[16] Zadeh A., Taylor D., Bertsos M., Tillman T., Nosoudi N., Bruce S. (2020). Predicting Sports Injuries with Wearable Technology and Data Analysis. Inf. Syst. Front..

[17] Evans S.A. (2022). The Biomechanics of Ice Hockey: Health and Performance Using Wearable Technology. J. Men’s Health.

[18] Crang Z.L., Duthie G., Cole M.H., Weakley J., Hewitt A., Johnston R.D. (2020). The Validity and Reliability of Wearable Microtechnology for Intermittent Team Sports: A Systematic Review. Sports Med..

[19] Rana M., Mittal V. (2020). Wearable Sensors for Real-Time Kinematics Analysis in Sports: A Review. IEEE Sens. J..

[20] Burland J.P., Outerleys J.B., Lattermann C., Davis I.S. (2021). Reliability of wearable sensors to assess impact metrics during sport-specific tasks. J. Sports Sci..

[21] Farì G., Latino F., Tafuri F., Dell’Anna L., Raele M.V., Fai A., Ranieri M. (2023). Shoulder Pain Biomechanics, Rehabilitation and Prevention in Wheelchair Basketball Players: A Narrative Review. Biomechanics.

[22] Hickey B.A., Chalmers T., Newton P., Lin C.-T., Sibbritt D., McLachlan C.S., Clifton-Bligh R., Morley J., Lal S. (2021). Smart Devices and Wearable Technologies to Detect and Monitor Mental Health Conditions and Stress: A Systematic Review. Sensors.

[23] Waqar A., Ahmad I., Habibi D., Hart N., Phung Q.V. (2021). Enhancing Athlete Tracking Using Data Fusion in Wearable Technologies. IEEE Trans. Instrum. Meas..

[24] Rangasamy K., As’ari M.A., Rahmad N.A., Ghazali N.F., Ismail S. (2020). Deep learning in sport video analysis: A review. TELKOMNIKA (Telecommun. Comput. Electron. Control.).

[25] Stein M., Janetzko H., Lamprecht A., Breitkreutz T., Zimmermann P., Goldlucke B., Schreck T., Andrienko G., Grossniklaus M., Keim D.A. (2017). Bring It to the Pitch: Combining Video and Movement Data to Enhance Team Sport Analysis. IEEE Trans. Vis. Comput. Graph..

[26] Pipkin A., Kotecki K., Hetzel S., Heiderscheit B. (2016). Reliability of a Qualitative Video Analysis for Running. J. Orthop. Sports Phys. Ther..

[27] Della Villa F., Buckthorpe M., Grassi A., Nabiuzzi A., Tosarelli F., Zaffagnini S., Della Villa S. (2020). Systematic video analysis of ACL injuries in professional male football (soccer): Injury mechanisms, situational patterns and biomechanics study on 134 consecutive cases. Br. J. Sports Med..

[28] Theodoropoulos J.S., Bettle J., Kosy J.D. (2020). The use of GPS and inertial devices for player monitoring in team sports: A review of current and future applications. Orthop. Rev..

[29] E Johansson R., Adolph S.T., Swart J., I Lambert M. (2020). Accuracy of GPS sport watches in measuring distance in an ultramarathon running race. Int. J. Sports Sci. Coach..

[30] Pino-Ortega J., Oliva-Lozano J.M., Gantois P., Nakamura F.Y., Rico-González M. (2022). Comparison of the validity and reliability of local positioning systems against other tracking technologies in team sport: A systematic review. Proc. Inst. Mech. Eng. Part P J. Sports Eng. Technol..

[31] Rico-González M., Arcos A.L., Clemente F.M., Rojas-Valverde D., Pino-Ortega J. (2020). Accuracy and Reliability of Local Positioning Systems for Measuring Sport Movement Patterns in Stadium-Scale: A Systematic Review. Appl. Sci..

[32] Luteberget L.S., Gilgien M. (2020). Validation methods for global and local positioning-based athlete monitoring systems in team sports: A scoping review. BMJ Open Sport Exerc. Med..

[33] Kupperman N., Hertel J. (2020). Global Positioning System–Derived Workload Metrics and Injury Risk in Team-Based Field Sports: A Systematic Review. J. Athl. Train..

[34] Huggins R.A., Giersch G.E., Belval L.N., Benjamin C.L., Curtis R.M., Sekiguchi Y., Peltonen J., Casa D.J. (2020). The Validity and Reliability of Global Positioning System Units for Measuring Distance and Velocity During Linear and Team Sport Simulated Movements. J. Strength Cond. Res..

[35] Waqar A., Ahmad I., Habibi D., Phung Q.V. (2021). Analysis of GPS and UWB positioning system for athlete tracking. Meas. Sens..

[36] Larsson P. (2003). Global Positioning System and Sport-Specific Testing. Sports Med..

[37] Le Flao E., Siegmund G.P., Borotkanics R. (2022). Head impact research using inertial sensors in sport: A systematic re-view of methods, demographics, and factors contributing to exposure. Sports Med..

[38] Taborri J., Keogh J., Kos A., Santuz A., Umek A., Urbanczyk C., Rossi S. (2020). Sport biomechanics applications using inertial, force, and EMG sensors: A literature overview. Appl. Bionics Biomech..

[39] Pajak G., Krutz P., Patalas-Maliszewska J., Rehm M., Pajak I., Dix M. (2022). An approach to sport activities recognition based on an inertial sensor and deep learning. Sens. Actuators A Phys..

[40] Moher D., Liberati A., Tetzlaff J., Altman D.G., Altman D., Antes G., Atkins D., Barbour V., Barrowman N., Berlin J.A. (2009). Pre-ferred reporting items for systematic reviews and meta-analyses: The PRISMA statement (Chinese edition). Chin. J. Intergr. Med..

[41] McKenzie J.E., Brennan S.E., Ryan R.E., Thomson H.J., Johnston R.V., Thomas J., Higgins J.P.T., Thomas J., Chandler J., Cumpston M., Li T., Page M.J., Welch V.A. (2023). Defining the criteria for including studies and how they will be grouped for the synthesis. Cochrane Handbook for Systematic Reviews of Interventions, Version 6.4.

[42] Downs S.H., Black N. (1998). The feasibility of creating a checklist for the assessment of the methodological quality both of randomised and non-randomised studies of health care interventions. J. Epidemiol. Community Health.

[43] Malan M., Dawson B., Goodman C., Peeling P. (2010). Effect of heat exposure on thermoregulation and hockey-specific response time in field hockey goalkeepers. J. Sci. Med. Sport.

[44] Polglaze T., Dawson B., Hiscock D.J., Peeling P. (2015). A Comparative Analysis of Accelerometer and Time–Motion Data in Elite Men’s Hockey Training and Competition. Int. J. Sports Physiol. Perform..

[45] Vescovi J.D., Frayne D.H. (2015). Motion Characteristics of Division I College Field Hockey: Female Athletes in Motion (FAiM) Study. Int. J. Sports Physiol. Perform..

[46] Sunderland C.D., Edwards P.L. (2017). Activity Profile and Between-Match Variation in Elite Male Field Hockey. J. Strength Cond. Res..

[47] Perrotta A.S., Held N.J., Warburton D.E. (2017). Examination of internal training load parameters during the selection, preparation and competition phases of a mesocycle in elite field hockey players. Int. J. Perform. Anal. Sport.

[48] Morencos E., Romero-Moraleda B., Castagna C., Casamichana D. (2018). Positional Comparisons in the Impact of Fatigue on Movement Patterns in Hockey. Int. J. Sports Physiol. Perform..

[49] Casamichana D., Morencos E., Romero-Moraleda B., Gabbett T.J. The Use of Generic and Individual Speed Thresholds for Assessing the Competitive Demands of Field Hockey. 2018, 17, 366–371.

[50] Kim T., Cha J.-H., Park J.-C. (2016). Association between in-game performance parameters recorded via global positioning system and sports injuries to the lower extremities in elite female field hockey players. Clust. Comput..

[51] Vinson D., Gerrett N., James D.V.B. (2018). Influences of Playing Position and Quality of Opposition on Standardized Relative Distance Covered in Domestic Women’s Field Hockey: Implications for Coaches. J. Strength Cond. Res..

[52] Vescovi J.D., Klas A. (2018). Accounting for the warm-up: Describing the proportion of total session demands in women’s field hockey—Female Athletes in Motion (FAiM) study. Int. J. Perform. Anal. Sport.

[53] Perrotta A.S., Warburton D.E.R. (2018). A comparison of sessional ratings of perceived exertion to cardiovascular indices of exercise intensity during competition in elite field hockey players. Biomed. Hum. Kinet..

[54] Polglaze T., Dawson B., Buttfield A., Peeling P. (2018). Metabolic power and energy expenditure in an international men’s hockey tournament. J. Sports Sci..

[55] Chesher S.M., Netto K.J., Appleby B.B., Jacques A., Wild C.Y. (2019). Deceleration characteristics of elite Australian male field hockey players during an Olympic tournament. J. Sci. Med. Sport.

[56] Warman G.E., Cole M.H., Johnston R.D., Chalkley D., Pepping G.-J. (2019). Using Microtechnology to Quantify Torso Angle During Match-Play in Field Hockey. J. Strength Cond. Res..

[57] Morencos E., Casamichana D., Torres L., Romero-Moraleda B., Haro X., Rodas G. (2019). Kinematic demands of interna-tional competition in women’s field hockey. Apunt. Educ. Física Deport..

[58] McGuinness A., Malone S., Hughes B., Collins K., Passmore D. (2019). Physical Activity and Physiological Profiles of Elite International Female Field Hockey Players Across the Quarters of Competitive Match Play. J. Strength Cond. Res..

[59] Harry K., Booysen M.J. (2020). Faster Heart Rate Recovery Correlates With High-Intensity Match Activity in Female Field Hockey Players—Training Implications. J. Strength Cond. Res..

[60] Romero-Moraleda B., Morencos-Martínez E., Torres-Ronda L., Casamichana D. (2020). Analysis of congested schedule on competition external load in field hockey. RICYDE. Rev. Int. Cienc. Del Deport..

[61] McGuinness A., McMahon G., Malone S., Kenna D., Passmore D., Collins K. (2020). Monitoring Wellness, Training Load, and Running Performance During a Major International Female Field Hockey Tournament. J. Strength Cond. Res..

[62] McGuinness A., Kenna D., Grainger A., Collins K. (2021). Investigating the Effect of Individual Rotations on the Physical and Physiological Performance in Elite Female Field Hockey Players. Appl. Sci..

[63] Ihsan M., Yeo V., Tan F., Joseph R., Lee M., Aziz A.R. (2021). Running Demands and Activity Profile of the New Four-Quarter Match Format in Men’s Field Hockey. J. Strength Cond. Res..

[64] McGuinness A., Passmore D., Malone S., Collins K. (2022). Peak Running Intensity of Elite Female Field Hockey Players during Competitive Match Play. J. Strength Cond. Res..

[65] Lin L., Ji X., Zhang L., Weng H., Wang X. (2023). Peak Running, Mechanical, and Physiological Demands of Professional Men’s Field Hockey Matches. J. Hum. Kinet..

[66] Sterne J.A.C., Savović J., Page M.J., Elbers R.G., Blencowe N.S., Boutron I., Cates C.J., Cheng H.Y., Corbett M.S., Eldridge S.M. (2019). RoB 2: A revised tool for assessing risk of bias in randomised trials. BMJ.

[67] Malone J.J., Barrett S., Barnes C., Twist C., Drust B. (2019). To infinity and beyond: The use of GPS devices within the football codes. Sci. Med. Footb..

[68] Silva A.F., Oliveira R., Cataldi S., Clemente F.M., Latino F., Badicu G., Greco G., Leão C., Bonavolontà V., Fischetti F. (2022). Weekly Variations of Well-Being and Interactions with Training and Match Intensities: A Descriptive Case Study in Youth Male Soccer Players. Int. J. Environ. Res. Public Health.

[69] la Torre M.E., Monda A., Messina A., de Stefano M.I., Monda V., Moscatelli F., Tafuri F., Saraiello E., Latino F., Monda M. (2023). The Potential Role of Nutrition in Overtraining Syndrome: A Narrative Review. Nutrients.

[70] Latino F., Greco G., Fischetti F., Cataldi S. Multilateral training improves body image perception in female adolescents. Proceedings of the Spring Conferences of Sports Science.

[71] Aughey R.J. (2011). Applications of GPS technologies to field sports. Int. J. Sport Physiol..

[72] Latino F., Tafuri F. (2023). Physical Activity and Academic Performance in School-Age Children: A Systematic Review. Sustainability.

[73] Latino F., Cataldi S., Fischetti F. (2021). Effects of an 8-Week Yoga-Based Physical Exercise Intervention on Teachers’ Burnout. Sustainability.

[74] Latino F., Tafuri F., Saraiello E., Tafuri D. (2023). Classroom-Based Physical Activity as a Means to Improve Self-Efficacy and Academic Achievement among Normal-Weight and Overweight Youth. Nutrients.

[75] Lee J., Wheeler K., James D.A. (2019). Wearable Sensors in Sport: A Practical Guide to Usage and Implementation.

[76] Nahavandi D., Alizadehsani R., Khosravi A., Acharya U.R. (2022). Application of artificial intelligence in wearable de-vices: Opportunities and challenges. Comput. Meth. Prog. Bio..

[77] Latino F., Cataldi S., Carvutto R., De Candia M., D’Elia F., Patti A., Bonavolontà V., Fischetti F. (2021). The Importance of Lipidomic Approach for Mapping and Exploring the Molecular Networks Underlying Physical Exercise: A Systematic Review. Int. J. Mol. Sci..

